# Physicochemical and Nutritional Characterization of Bran-Enriched Products

**DOI:** 10.3390/foods11050675

**Published:** 2022-02-25

**Authors:** Catrin Tyl, Alessandra Marti

**Affiliations:** 1 Faculty of Chemistry, Biotechnology and Food Science, Norwegian University of Life Science, 1433 Ås, Norway; 2 Department of Food, Environmental and Nutritional Sciences (DeFENS), Università degli Studi di Milano, Via Giovanni Celoria, 2, 20133 Milan, Italy

The incorporation of cereal bran or bran constituents can improve the nutritional profile of products and serve as a means to utilize milling by-products that otherwise may only go towards feed. While bran is an excellent vehicle for dietary fiber and phytochemicals with presumed bioactive function, its addition to formulations alters product properties.

Against this background, this Special Issue on “Physicochemical and Nutritional Characterization of Bran-Enriched Products” highlights numerous approaches related to the design of bran-enriched products. The studies of this Special Issue focused on a wide range of materials, including whole grains (ancient wheats and sorghum), bran from wheat, oat, and the perennial grain intermediate wheatgrass, as well as isolated arabinoxylans.

While refined bread wheat flour is still the most important and versatile material for baking, there is interest in exploring various types of whole grain flours for this purpose. Kulathunga et al. [1] evaluated kernel quality and chemical composition of genotypes from einkorn, emmer, spelt, and hard red spring. The samples were cultivated in the same year and location, with identical environmental conditions. Kernel quality traits, including test weight, 1000 kernel weight, and kernel hardness, of ancient wheats were different to hard red spring wheat, likely due to their kernel shape and endosperm microstructure. Einkorn and emmer were identified as extra soft- and hard-textured kernels, respectively, while both medium-soft and hard genotypes were observed in spelt. Ancient wheats were characterized by lower average protein and higher crude fat contents than hard red spring wheat, suggesting the need for further studies on lipid stability during storage and processing.

Two main reformulation approaches can be taken to increase the fiber level in cereal-based products: using whole-meal (i.e., flour containing bran, germ and endosperm in the same proportions as found in the grain) or including bran previously separated from the endosperm through milling. When the first approach is selected, wholemeal flour can be produced by either single-stream milling (generally using stone mills) or by multiple-stream milling (using roller mills) with the recombination of flour fractions. Rumler et al. [2] applied both milling techniques to sorghum to highlight that despite distinctive differences in kernel morphology, wholemeal sorghum flours can successfully be produced with equipment designed for wheat milling. Compositional differences between the samples obtained through roller or stone-milling (i.e., higher protein, fiber, and total phenolic content in the former) were attributed to differences in milling yields. The milling system also affected the hydration properties: whole sorghum flour obtained from the recombination of the roller mill fractions showed a lower water absorption and higher water solubility, properties that can affect flour behavior during processing.

The importance of bran particle size on product quality is increasingly being recognized as a crucial parameter that requires optimization. However, different products may benefit from different mean particle sizes and distributions, as highlighted by both Huang et al. [3] and Molina et al. [4]. In the work of Huang et al. [3], material from three classes of wheat and four particle size distributions of bran was reconstituted to create wholewheat flour for the production of northern-type steamed bread. Compared to material with D(50) values of 53 and 74 μm, bran particle sizes of 105 μm and 125 μm produced whole-wheat steamed bread with a larger specific volume, brighter color, and softer texture. Molina et al. [4] pointed out that the incorporation of fine bran particles (with only 4% of particles >500 μm) into rotary-molded biscuit recipes did not impair molding, a critical operation in this biscuit type. Moreover, the fracturability of biscuits was practically unaltered compared to biscuits prepared with refined flour. Finer bran fractions produced a discontinuous and compact structure that increased the overall strength of dough compared to coarse bran particles (with 72% of particles >500 μm). The authors proposed that micropores present in coarse wheat bran may act as capillaries that retain water before but release water during baking, thereby increasing the degree of gelatinization.

The addition of bran to flour-based products can interfere with the hydration of protein and starch, as well as their aggregation and gelatinization, respectively. This negatively affects the structuring processes crucial for achieving the desired shapes and textures of the final products. Several studies in this Special Issue assessed strategies to modify bran constituents and improve functional attributes. Using glass beads as inert fillers, Renzetti et al. [5] elucidated how chemical and physical interactions contribute to the effect of oat or wheat bran and their soluble and insoluble fiber fractions on dough and bread properties. Based on their results, the authors proposed that the impact of different bran and fiber types on bread volume and texture relates to the interplay between water binding (mainly influenced by the insoluble bran fraction), and the plasticizing properties of the aqueous phase (mainly affected by the soluble bran fraction). The soluble oat bran fraction, rich in β-glucans, impaired bread quality more than wheat bran solubles. Soluble and insoluble fractions either augmented the viscous or elastic behavior, respectively. Structure setting is influenced by starch gelatinization and gluten aggregation, processes that were both impacted by fiber constituents, most importantly the β-glucans. The findings of Renzetti et al. [5] are also interesting in relation to the results from Molina et al. [4]. In rotary-molded biscuits, wheat bran had the greatest impact on dough firmness while arabinoxylans—the most important non-starch polysaccharides in whole wheat flour—had the greatest impact on the elastic response [4].

To overcome the negative impact of bran on product properties, several treatments have been proposed. Extrusion cooking has the potential to beneficially affect bran’s functional and nutrition-related properties. Bran extrusion induces a transition from a very dense to a more open matrix. Roye et al. [6] investigated whether wheat bran properties reflective of such structural disintegration are affected similarly when extrusion is performed at pilot vs. industrial scale. The degradation of bran structure was promoted the most by using an industrial-scale extruder at low moisture contents, as a higher specific mechanical energy could be achieved this way. On the other hand, bran processed using an industrial-scale extruder at high barrel temperatures exhibited increased interaction with water. The authors suggested that such modifications of bran structure could impact physiological effects such as the fermentability of dietary fiber.

Another strategy to modify bran is via incubation with enzymes. Dai et al. [7] focused their efforts on intermediate wheatgrass, a perennial grain of interest due to its considerable root mass, which may help counteract environmental challenges associated with intensive cereal production. However, the combination of weak protein networks and high fiber contents limits its use in baked goods. Dai et al. [7] used bran pre-treatment with xylanase to modify the fiber population, resulting in increased expansion, evidenced by higher specific volumes and differences in crumb structure. The effects on dough and bread were partially counteracted by ascorbic acid, added as a dough strengthener that also improved surface appearance. While further product optimization and mechanistic studies are needed, the results suggest that suitable processing methods can open up possibilities for bread production with intermediate wheatgrass bran. 

Rosa-Sibakov et al. [8] combined enzymatic treatment (i.e., β-glucanase) with mechanical treatment (i.e., microfluidization) to improve the technological properties of oat bran concentrate intended for high-moisture food applications. Both treatments effectively decreased the molecular weight of β-glucans and the viscosity. The addition of treated samples to acid milk gels decreased syneresis and improved the water holding capacity. Gels with microfluidized samples had a firmer texture than the ones enriched with enzyme-treated samples. The overall modifications upon treatments encourage the utilization of oat bran in the development of β-glucan-rich, high-moisture food products.

Product formulation with oat bran was also performed by Onipe et al. [9]. The authors incorporated it into *magwinya*, a deep-fried food consumed in parts of sub-Saharan Africa, and compared its impact on *in vitro* starch digestibility and estimated glycemic index to products enriched with wheat bran. The incorporation of oat or wheat bran into dough, as well as batter preparations, caused a similar reduction in rapidly available glucose, as well as an increase in slowly available and unavailable glucose. The estimated glycemic index decreased in bran-enriched formulations, with wheat bran having the greatest effect. Overall, bran enrichment was shown to delay glucose release of *magwinya*, a popular main dish component of low-income populations.

A further product category covered in this Special Issue are extruded snacks. Tyl et al. [10] addressed the effects of extrusion cooking on bran’s compositional aspects, outlined how extrudate properties are affected when bran is added, and summarized literature that assessed optimum processing conditions. The review highlighted how research on bran modifications led to bran-enriched extrudates with improved structural characteristics. However, numerous unsolved questions remain, such as how consumer behavior and the communication of bran’s nutritional benefits influence the perception of bran-enriched extruded snacks.

Nutrition claims are intended to inform consumers about the nutritional characteristics of the product, but according to the study of Martini et al. [11], purchasing breakfast cereals carrying a “source of fiber” or “high in fiber” claim does not guarantee the acquisition of products with improved nutritional quality compared to products without such claims. The Authors suggested that consumers should be educated to carefully read the entire nutritional information reported on the labels as (i) several products did not report a fiber-related claim, despite containing sufficient fiber to allow for such types of claims; (ii) the overall differences in formulation may affect the nutritional composition of the product more than the presence of a certain amount of fiber added to meet claims.

The guest editors would like to thank all authors for their contributions to this Special Issue.
